# Supplementary material for: Evaluating the utility of amino acid similarity-aware kmers to represent TCR repertoires for classification
Source: PLoS Comput Biol. 2026 Apr 30;22(4):e1014211. doi: 10.1371/journal.pcbi.1014211 (PMC13132464; doi:10.1371/journal.pcbi.1014211)
Supplement: S1 Table — Chosen hyperparameters for XGBoost models including reg_lambda, max_depth and learning_rate and for logistic regression models limited to the regularisation hyperparameter C trained on the CMV training dastaset. (PDF) [file pcbi.1014211.s001.pdf]

**Model**	**Features**	**Encoding**	**s**	**reg_lambda**	**max_depth**	**learning_rate**	**C**
XGB	kmers			1.36	3	0.0837	
XGB	RA kmers	BLOSUM62	10	62.7	8	0.0853	
XGB	RA kmers	Atchley	9	27.4	9	0.0940	
XGB	kmer clusters	BLOSUM62		31.1	10	0.0887	
XGB	kmer clusters	Atchley		12.3	3	0.0700	
L1LR	kmers						0.0405
L1LR	RA kmers	BLOSUM62	11				0.0260
L1LR	RA kmers	Atchley	13				0.0386
L1LR	kmer clusters	BLOSUM62					0.0451
L1LR	kmer clusters	Atchley					0.350
