# Supplementary material for: Evaluating the utility of amino acid similarity-aware kmers to represent TCR repertoires for classification
Source: PLoS Comput Biol. 2026 Apr 30;22(4):e1014211. doi: 10.1371/journal.pcbi.1014211 (PMC13132464; doi:10.1371/journal.pcbi.1014211)
Supplement: S2 Table — Chosen hyperparameters for XGBoost models including reg_lambda, max_depth and learning_rate and for logistic regression models limited to the regularisation hyperparameter C trained on the CeD training dataset. (PDF) [file pcbi.1014211.s002.pdf]

**Model**	**Features**	**Encoding**	**s**	**reg_lambda**	**max_depth**	**learning_rate**	**C**
XGB	kmers			2.34	5	0.0899	
XGB	RA kmers	BLOSUM62	14	7.29	3	0.0206	
XGB	RA kmers	Atchley	12	36.7	4	0.0121	
XGB	kmer clusters	BLOSUM62		39.5	5	0.0686	
XGB	kmer clusters	Atchley		7.18	10	0.0719	
L1LR	kmers						0.783
L1LR	RA kmers	BLOSUM62	14				0.0897
L1LR	RA kmers	Atchley	18				0.0586
L1LR	kmer clusters	BLOSUM62					0.106
L1LR	kmer clusters	Atchley					0.405
