# Supplementary material for: Evaluating the utility of amino acid similarity-aware kmers to represent TCR repertoires for classification
Source: PLoS Comput Biol. 2026 Apr 30;22(4):e1014211. doi: 10.1371/journal.pcbi.1014211 (PMC13132464; doi:10.1371/journal.pcbi.1014211)
Supplement: S3 Table — Table shows full motifs with odds ratios and adjusted P-values. (PDF) [file pcbi.1014211.s003.pdf]

**rank**	**motif**	**odds ratio**	**P-value (adjusted)**
1	C[AST][AST][AST]	1.439	$1.12e-72$
2	[AST][AST][ILMV]G	1.49	$7.63e-23$
3	C[AST][AST][EKQR]	0.758	$9.17e-21$
4	[AST][AST][EKQR][EKQR]	0.710	$1.09e-18$
5	[DN][AST][EKQR][AST]	1.32	$2.04e-10$
6	G[FY][DN][EKQR]	1.89	$5.33e-09$
7	[AST][AST][ILMV][ILMV]	1.35	$8.03e-09$
8	[AST][AST][ILMV][AST]	1.23	$3.35e-06$
9	[EKQR][EKQR][FY][FY]	1.12	$3.94e-06$
1	[AST][AST]PG	1.41	$4.61e-06$
11	[EKQR][EKQR]G[EKQR]	0.565	$6.16e-06$
12	[AST][EKQR][EKQR][EKQR]	0.686	$5.78e-05$
13	[EKQR]G[EKQR][EKQR]	0.647	$1.76e-04$
14	[AST][ILMV]GG	1.45	$2.92e-04$
15	[EKQR][AST]G[EKQR]	0.686	$2.95e-04$
16	[AST][AST][EKQR][ILMV]	0.799	$3.40e-04$
17	C[AST][EKQR][AST]	0.240	$5.80e-04$
18	G[DN][AST][ILMV]	0.744	$6.82e-04$
19	G[ILMV][DN][AST]	1.536	$7.94e-04$
20	[AST][DN][AST][EKQR]	1.17	0.00100
21	[AST][AST]G[EKQR]	0.794	0.00100
22	[AST][AST][EKQR][AST]	0.852	0.00280
23	[AST][EKQR][EKQR]G	0.744	0.00349
24	G[EKQR]G[EKQR]	0.669	0.00404
25	[AST][EKQR][AST]P	0.571	0.00527
26	[EKQR][EKQR][EKQR][FY]	0.649	0.00542
27	[EKQR]G[EKQR][AST]	0.691	0.00824
28	[DN][AST][EKQR][FY]	1.14	0.00832
29	[EKQR][EKQR][EKQR]G	0.634	0.00964
30	P[ILMV]G[EKQR]	0.353	0.0110
31	[AST]G[EKQR]G	0.743	0.0143
32	[EKQR][AST][AST][AST]	0.648	0.0176
33	[EKQR][AST][EKQR][EKQR]	0.621	0.0211
34	[EKQR][ILMV]GG	0.545	0.0221
35	[EKQR][AST][ILMV]G	0.530	0.0233
36	[AST][EKQR][ILMV][AST]	0.735	0.0240
37	[AST][ILMV][DN][AST]	1.39	0.0297
38	[AST]G[EKQR][EKQR]	0.745	0.0333
39	[EKQR][EKQR][AST]G	0.690	0.0410
40	G[AST][FY][EKQR]	1.36	0.0429
41	[AST][EKQR][ILMV][EKQR]	0.672	0.0446
