# Supplementary material for: Evaluating the utility of amino acid similarity-aware kmers to represent TCR repertoires for classification
Source: PLoS Comput Biol. 2026 Apr 30;22(4):e1014211. doi: 10.1371/journal.pcbi.1014211 (PMC13132464; doi:10.1371/journal.pcbi.1014211)
Supplement: Attachment — Submitted filename: response_to_reviewers.docx [file pcbi.1014211.s008.docx]

We thank the reviewers for their constructive evaluation of our manuscript and insightful comments, which we believe have led to considerable improvement in the quality and clarity of our work. We have undertaken major revisions. Most notably, we performed a runtime experiment for the CMV classification problem, and for both CMV and CeD problems, included regularised logistic regression models and interpretation analysis, where kmers/motifs important to a model are inspected and compared to immune status-associated CDR3 sequences from the literature. Further, we have amended much of the existing text. Discussion concerning performance evaluation has been edited with care and a greater focus on testing results. We now respond to individual points in reviewers' feedback in blue.

# Reviewer 1

Reviewer #1: This paper focused on the role of reduced amino acid alphabets in kmer-based disease state prediction using T-cell receptor (TCR) repertoires. Two encoding methods, Atchley factors and BLOSUM62-based distance matrices, are used to cluster amino acids into reduced alphabets. This proposed method is evaluated against standard kmer approaches and existing kmer clustering methods. However, there are several limitations that should be addressed to improve the completeness and robustness of the work.

## Major comments

1. **Feature redundancy and model generalizability: The feature importance analysis reveals that only a small fraction of kmer features contribute meaningfully to classification, indicating that most features are redundant. The models may rely heavily on a few high-frequency kmers, potentially limiting its generalization. The authors should consider applying feature selection or dimensionality reduction to mitigate overfitting.**

We thank reviewer 1 for raising this important point. We use regularised models, XGBoost and logistic regression with a LASSO penalty, which are considered to be embedded feature selection methods. These automatically remove features from the model, and indeed, most of our kmer features are redundant. We believe that current regularization strategies result in good model generalisation, as demonstrated by the consistency between validation and testing performance. In addition, we applied scaling to zero mean, unit standard deviation to each feature which should mitigate heavy reliance on high frequency kmers. These details are contained in Methods: Classification methods: XGBoost models; Regularised logistic regression models; Evaluation of TCR repertoire classification models.

2. **Potential information leakage due to sample split strategy: The training and testing sets are split by sample/cohort, but it is unclear whether the sequences across these sets are truly independent. Overlap in sequence regions may result in shared kmers between training and test sets, which could cause inflated performance. The authors should consider alternative data partitioning strategies, such as clustering sequences based on similarity.**

We agree with reviewer 1 in that information leakage should be avoided with great care, but ultimately maintain that we have avoided leakage in our training and testing setup to the greatest extent we could achieve, as evidenced by code provided with the manuscript. Our approach aims to classify samples, and therefore our training and testing strategy is based on a realistic scenario whereby whole samples are unseen. We might expect to see the same TCR sequence in two individuals in the general population, and we would certainly expect the same kmer to be observed across two individuals.

## Minor comments

 1. **Limited model comparison: Only XGBoost is used for classification. A comparison with other traditional classifiers such as Random Forest, Support Vector Machine (SVM), or Logistic Regression would strengthen the analysis and provide insight into whether the performance gains are model-specific.**

We have included regularised (with L1-norm, or LASSO penalty) logistic regression results, and thank the reviewer for their valuable suggestion. This additional analysis provides further understanding of the model-specific effect of a reduced alphabet as mentioned by the reviewer, as well as the benefit of a non-linearity in a kmer-based TCR model. This additional analysis can be found in Results: CMV classification problem from line 153, and CeD classification problem from line 293.

**2 . Lack of efficiency analysis: Although the reduced alphabet method is claimed to be more efficient and flexible than existing kmer clustering methods, no empirical or theoretical comparison (e.g., runtime or complexity) is provided. Including such analysis would help substantiate these claims.**

An efficiency analysis was undertaken, and we reflect that it strengthens the argument in the manuscript greatly, and enables us to make clearer conclusions. This analysis is detailed in section Results: CMV XGBoost model runtime comparison starting line 192, and involves training on the CMV dataset over a harmonised range of amino acid similarity hyperparameters for kmer clustering and our reduced alphabet method.

**3. Representation limitations of kmer features: Compared to deep learning methods (e.g., CNNs or attention-based models), kmer-based representations may suffer from loss of contextual information, limited adaptability, and poor transferability across tasks. The authors should consider discussing the relative strengths and weaknesses of their approach in comparison to more expressive models.**

In the introduction, lines 46-52, we discuss deep learning methods in more detail. Deep learning methods are certainly more expressive and are uniquely suitable for some tasks such as identifying objects within images. Operating within a data-poor environment with labelled whole TCR repertoires, we focus on kmer-based representations, but acknowledge good work undertaken by others in deep learning methods for TCR repertoire classification. We thank reviewer for raising this point, as this discussion adds valuable context for the work undertaken. We also mention deep learning in the context of interpretability in Discussion lines 459-461, highlighting greater inherent interpretability of XGBoost in comparison.

# Reviewer 2

Reviewer #2: The authors present an approach for T-cell receptor (TCR) repertoire classification using amino acid similarity-aware k-mer representations. They used the reduced alphabets for computational efficiency and compared it against standard k-mers and k-mer clustering approaches on cytomegalovirus (CMV) infection and coeliac disease (CeD) datasets. Althouth the work is interesting, several major concerns need to be addressed.

1. **The proposed reduced alphabet approach shows only small improvement over standard k-mers. In particular, for CeD classification using test datasets, standard k-mers actually outperform the proposed method (AUROC 0.934 vs 0.860). The authors should clarify that the proposed method is better than the basic k-mer method and others.**

The reviewer is correct in that standard kmers outperform the reduced alphabet kmers for the CeD classification problem in testing. We have rewritten parts of the abstract, results and discussion to reflect our focus on testing results rather than validation, and moved validation performance tables to supplementary. We believe that the manuscript has value as it may provide researchers with a fair evaluation of kmer representations that include amino acid similarity information, and presents an efficient method for incorporating user-defined amino acid similarity into a kmer representation.

**2. Furthermore, it would helpful to provide runtime comparisons between your proposed method and others to support the computational efficiency.**

As described in response to reviewer 1 who also made this very helpful suggestion, we performed an efficiency analysis which is detailed in section Results: CMV XGBoost model runtime comparison starting line 192, and involves training on the CMV dataset over a harmonised range of amino acid similarity hyperparameters for kmer clustering and our reduced alphabet method.

3. **The authors used XGBoost with a fixed hyperparameter for the classifiction. How did the hyperparameters are chosen? They should present how they found the optimal hyperparameters in details.**

The XGBoost hyperparameters were indeed fixed. They were chosen and fixed based on sensible values either equivalent or close to values used by Katayama and Kobayashi Front Immunol 2022 (doi:10.3389/fimmu.2022.797640) in their ablation study with no hyperparameter search, as described in Methods: Classification methods: XGBoost models. We did not optimise any hyperparameters except the amino acid similarity threshold (reduced alphabet size), which is discussed in Methods: Classification Methods: Optimisation of reduced alphabet size.

**4. In addition, the authors should show and compare the experimental results with other classfiers (Random Forests, SVMs, Artificial Neural Networks, etc.).**

We are in agreement with reviewer 2 and reviewer 1 that the manuscript would benefit from inclusion of an additional classification method. We chose to include regularised logistic regression, which, as a linear classifier, provides a contrast to the non-linear XGBoost method for which results are shown in Results: CMV classification problem from line 153, and CeD classification problem from line 293.

5. **Why did the authors downsample the datasets at the experiments? Even though they already provided some explanation, a more detailed and comprehensive discussion is needed for clarity. If possible, please show the experimental results without the downsampling to clarify the impact of the decision.**

We welcome the opportunity to discuss downsampling in more detail. We have expanded on our initial explanation of downsampling in Results: CMV classification problem, starting at line 142. Here, we also provide some evidence from the literature that our methods are likely to be robust to downsampling. In addition, a row has been added to each of tables 1 and 4 showing the mean, minimum and maximum number of TCR sequences per repertoire before downsampling to clarify the effect of the downsampling operation. We regard downsampling as an important preprocessing step which should result in a model that more closely reflects biology rather than technical variation, such as degradation of DNA in samples or amount of material sampled.

**6. The XGBoost model uses thousands of features, making interpretation challenging. Although the authors performed the feature importance analyses (Figures 3 and 6, S1-S2), it provides little biological understanding. The authors should include some biological insights clearly. For example, they should show clearly whether the identified motifs have some direct relationships to the previously known TCR-antigen interactions.**

The reviewer is correct: interpretation of our models is challenging. We now make steps towards addressing this challenge by including comparisons of kmers/motifs in known CMV-specific and CeD associated CDR3 sequences from the literature in sections Results: Interpretation of CMV classification models from line 222 and Results: Interpretation of CeD classification models from line 304. We are able to identify a CeD-associated 4mer motif from Han et al. PNAS 2013 (doi:10.1073/pnas.1311861110) in our XGBoost features, and show that it could potentially be extended to a 6mer. This demonstrates that our model is capable of identifying known associations between sequences and CeD as well as potentially identifying new ones.

**7. The sample size of CeD datasets is limited. Is the number of samples sufficient to train the model effectively? In addition, the CeD datasets exhibit a significant imbalance between the positive and negative samples than CMV datasets. The authors should provide a clear and detailed discussion on why both the small number of samples and the imbalanced datasets were not significant problems if they claim this to be the case.**

We acknowledge that our sample sizes are small for the CeD classification problem, and that we do indeed have a class imbalance in the CeD training data. We include discussion of these limitations in Discussion starting line 393. To summarise, we use metrics that are not sensitive to class imbalance such as accuracy, and use a class weighing strategy in XGBoost and logistic regression models to mitigate the effects of sample size and class imbalance. It is also mentioned that testing performance is less certain as an estimate due to sample size. The immune repertoire in CeD is relatively well studied with a small number of sequences known to be associated with the disease; we have also included comparison to these sequences in Results: CeD model interpretation starting line 304 which has provided an additional level of validation for our model.

# Reviewer 3

Reviewer #3: The immune system keeps a record of past infections and current immune state. Advances in sequencing now allow large-scale profiling of the adaptive immune receptor repertoire, which encodes this record. However, linking immune repertoires to functional outcomes remains a major challenge. Machine learning offers a promising path forward, yet the immense diversity of immune receptors often necessitates some form of coarse-graining of sequence information before model training.

This paper evaluates the use of kmers derived from reduced amino acid alphabets as coarse-grained representations of immune repertoires. Kmer-based classification remains a valuable baseline approach, even in the era of deep learning. Extending this method through reduced amino acid alphabets is an interesting idea with potential to advance the field. However, the current manuscript has several important limitations.

## Major concerns:

**1. The performance improvements obtained using reduced amino acid alphabets are not statistically significant in cross-validation. For instance, AUROC scores in CMV classification (Table 2: 0.75 ± 0.03 vs. 0.77 ± 0.04) and CeD classification (Table 5) show no clear evidence that reduced alphabet representations outperform baseline. Similarly, there is no consistent advantage of BLOSUM-based alphabets over Atchley-based ones. Can the authors identify a specific scenario where reduced alphabets yield a meaningful benefit? If not, the abstract and discussion should be revised to more accurately reflect the evidence provided.**

This is correct, and we thank reviewer 3 for raising this important point. We have made significant changes to the abstract and discussion, focusing mostly on testing results as hold-out testing provides a higher standard of evidence for model performance. We believe the revised manuscript provides a clearer reasoning to justify dissemination of the reduced alphabet methodology as a result. With new understanding of functional similarity of amino acids in a TCR e.g. Henderson et al. PNAS 2024 (doi:10.1073/pnas.2408696121), the reduced alphabet method offers a more efficient approach to integrate this valuable information into a kmer-based TCR repertoire classification model compared to kmer clustering.

**2. All reported models perform substantially worse on the CMV dataset than the method introudced by Emerson et al. (Nature Genetics 2017, doi:10.1038/ng.3822), who first described this dataset. The manuscript should address this discrepancy directly and more clearly contextualize the contribution of the present work relative to methods with demonstrated higher performance.**

The reviewer makes an insightful point, and we take the opportunity to discuss the results by Emerson et al. in more detail and in context of our own in Discussion, starting line 377.

## Minor concerns:

**3. The introduction states that "the underlying relationships between amino acids that may lead to shared function are unknown". However, recent works have begun to address this gap, for example Henderson et al. PNAS 2024 (doi:10.1073/pnas.2408696121) compared the ability of different reduced amino acid alphabets to capture functional similarity using known TCR–pMHC pairings.**

We thank the reviewer for suggesting an interesting read, and cite this relevant paper in our Introduction, line 61, and Discussion, line 440.

5. **On lines 119-124, does the similarity threshold refers to the hierarchical clustering of Atchley factors or BLOSUM62? At each similarity threshold a fixed integer-sized reduced alphabet is obtained, so it was unclear what is meant with the effective number of 3.16.**

On lines 119-124 we originally referred to any generic reduced alphabet. We agree that this point was unclear, and therefore have clarified and rephrased on lines 127-137. We instead focus on conceptualising amino acid similarity roughly as a function of group size, be it of motifs of kmers or clusters of kmers, for a generic reduced alphabet and conclude that an alphabet of size 3 is most like clustering into 100 groups of kmers if the majority of possible kmers are observed.
