# Supplementary material for: Evaluating the utility of amino acid similarity-aware kmers to represent TCR repertoires for classification
Source: PLoS Comput Biol. 2026 Apr 30;22(4):e1014211. doi: 10.1371/journal.pcbi.1014211 (PMC13132464; doi:10.1371/journal.pcbi.1014211)
Supplement: Attachment — Submitted filename: response_to_reviewers_2.docx [file pcbi.1014211.s009.docx]

We thank the reviewers again for their constructive and valuable feedback. We have revised the manuscript to reflect a new set of results for both datasets obtained with hyperparameter optimisation in combination with our previously methodology. Although no specific revisions were suggested by reviewer 1, we agree that software should be maintained for the purposes of reproducibility, and plan to release updated software prior to publication. We now respond to individual points in reviewers' feedback in blue.

***Reviewer #2: The authors have adequately addressed most of the previous concerns. However, regarding comment #3, the justification for using fixed hyperparameters remains unclear. While the authors provided additional explanation in their response, I recommend that the authors conduct hyperparameter tuning to identify optimal parameters, which would strengthen the methodological rigor of the study.***

We agree with the reviewer that identification of optimal hyperparameters would strengthen any conclusions made about the five kmer-based representations we explored. As a result of performing hyperparameter optimisation with Bayesian optimisation for XGBoost and logistic regression models, differing hyperparameters were set for different feature types, seen in table S1 and table S2. This suggests that optimal hyperparameters may differ depending on kmer representation used, which leads us to believe that hyperparameter optimisation is indeed important to the rigour of this work.

We obtained new testing results for both CMV and CeD classification problems. Although our main results have changed, ultimately our conclusions are largely the same as the previous submission. These main results are shown in tables 2 and 4, which now include XGBoost and logistic regression results combined. To reflect that we moved straight to testing and training, validation results in tables S1 and S2 were replaced with the chosen hyperparameters for each of the 5 feature types for XGBoost and logistic regression. In addition, we replaced previous figures 2 and 3 with figure 2 and figures 10 and 11 with figure 9 which show the mean AUROC for each reduced alphabet size evaluated in cross-validation.

Since for the CeD classification problem our top-performing model is now XGBoost with BLOSUM62 reduced alphabet kmers, we interpreted this model instead of the standard kmer feature model. However, the biological insights obtained are rather similar. Figures 5 (previously 6), 6 (previously 7), 7 (previously 8), 10 (previously 12), 11 (previously 13), 12 (previously 14), S1, S2 and S3 have all changed to reflect the new final models. Extended motif list from figure 7 in table S3 has also been updated.

We edited the text to reflect the change in hyperparameter setting methodology. On lines 157-161 we added:

*“The five representations, and up to 20 different alphabets within the reduced alphabet representations, may require different model hyperparameter settings such as regularisation strength in order to train an optimal model. To tailor models to each representation, we set XGBoost hyperparameters reg_lambda, max_depth and learning_rate and logistic regression hyperparameter C, using Bayesian optimisation.”*

In Methods, we also added section Classification methods: Model hyperparameter optimisation:

*“Model hyperparameters are set using Optuna which implements Bayesian optimisation [69]. For each classification model, training data is split into a training fold with 80% of samples, and a validation fold of 20% of samples. A model is iteratively trained on the training fold with variable hyperparameters, and the validation fold is used to calculate AUROC as the objective for Bayesian optimisation to maximise. We performed 50 trials for each model. For XGBoost hyperparameters, ranges from which they are trialled are 1− 100 for reg_lambda, 3− 10 for max_depth, and 0.01− 0.1 for learning_rate. Logistic regression hyperparameter C is set within range 0.01− 1., which is consistent with reg_lambda given that they are inversely equivalent. For all hyperparameters except max_depth which was set as an integer, we use log= True with float type.”*

To Methods section Classification methods: Optimisation of reduced alphabet size we added:

*“Additionally, model hyperparameters are optimised for each alphabet size, resulting in a nested validation strategy.”*

To Methods section Classification methods: XGBoost models we added:

*“For all XGBoost models, we set scale_pos_weight of negative to positive class ratio as recommended in the documentation to reduce the effects of class imbalance. For evaluation of classification performance we employed a hyperparameter optimisation strategy for reg_lambda, max_depth, and learning_rate. In the runtime experiment, we set reg_lambda = 1, max_depth = 10 and learning_rate = 0.1. Otherwise, all other defaults were used.”*

And to Methods section Classification methods: Regularised logistic regression models we added:

*“We used a hyperparameter optimisation strategy to set C, and otherwise set penalty= l1, solver= liblinear, class_weight= balanced and all other defaults.”*

Minor edits to reflect new results based only on training and testing are shown in the markup document and listed as follows:

One sentence edited in each of the abstract and author summary

Results

- lines 157-161
- lines 171-172
- lines 176-190
- line 237
- lines 278-279
- lines 282-283
- lines 284-293
- lines 298-299
- lines 308-311
- lines 315-318
- lines 324-330
- lines 337-338
- lines 340-344
- lines 347-349

Discussion

- lines 357-359
- lines 370-371
- lines 381-389
- line 405
- lines 441-447
- lines 462-467
- line 472
