# Supplementary material for: Evaluating the utility of amino acid similarity-aware kmers to represent TCR repertoires for classification
Source: PLoS Comput Biol. 2026 Apr 30;22(4):e1014211. doi: 10.1371/journal.pcbi.1014211 (PMC13132464; doi:10.1371/journal.pcbi.1014211)
Supplement: Attachment — Submitted filename: response_r3.docx [file pcbi.1014211.s010.docx]

Dear Dr Zhu and Professor Tanaka,

On suggestion of reviewer 2 we have made additions to the GitHub repository <https://github.com/hannrko/enc_kmer_tcr_models>. We thank the reviewer for their useful suggestions for enhancing reproducibility of this work.

To summarise, we have included a *README.md*, an example script *example_enc_kmer_tcr_model.py*, a *requirements.txt* file, and made changes to the python code to reflect the most recent version of the manuscript. In more detail, we have:

- Updated *clas_model_wrappers.py* to include versions of XGBoost and logistic regression with Bayesian optimistion, including 80/20 train/validation splitting to evaluate each trial (controlled by test_prop=0.2 default argument)
- Added timing option used in first round of revisions, involving changes in files *aa_alph_redu/aa_alph_reducer.py*, *aa_alph_redu/ml_utils/ml_eval.py*, *bench_model.py*, *clas_model_wrappers.py* and *kmer_cluster.py*
- Added *run_enc_kmer_tcr_model.py* which runs training and testing for given kmer matrices and settings, and optionally saves results
- Added example script *example_enc_kmer_tcr_model.py* which randomly generates 4mer matrices for training and testing and runs an Atchley factor reduced alphabet XGBoost model with Bayesian optimisation
